# Subregional volume reduction of the cholinergic forebrain in subjective cognitive decline (SCD)

**DOI:** 10.1016/j.nicl.2018.101612

**Published:** 2018-11-27

**Authors:** Lukas Scheef, Michel J. Grothe, Alexander Koppara, Marcel Daamen, Henning Boecker, Hans Biersack, Hans H. Schild, Michael Wagner, Stefan Teipel, Frank Jessen

**Affiliations:** a Department of Radiology, University of Bonn, Germany; b German Center for Neurodegenerative Diseases (DZNE), Rostock, Germany; c Department of Psychiatry, University of Bonn, Germany; d German Center for Neurodegenerative Diseases (DZNE), Bonn, Germany; e Department of Nuclear Medicine, University of Bonn, Germany; f Department of Psychosomatic Medicine, University of Rostock, Rostock, Germany; g Department of Psychiatry, Medical Faculty, University of Cologne, Germany

**Keywords:** Basal forebrain, Subjective cognitive decline, Nucleus basalis Meynert, Glucose metabolism, Precuneus, Alzheimer

## Abstract

Subjective cognitive decline (SCD) patients are considered as a risk population for preclinical Alzheimer's Disease (AD). Supporting this idea, previous studies in SCD populations report subtle alterations in various cognitive and neuroimaging biomarkers that are typically affected during AD progression. To extend these observations, the present study examined whether SCD patients show atrophy of cholinergic basal forebrain nuclei (chBFN), analogous with recent findings in prodromal and clinical AD patients. We assessed volume reductions of the chBFN in 24 SCD subjects compared to 49 matched controls on 3D-T1-weighted MR images based on a postmortem derived atlas. Furthermore, we assessed whether chBFN atrophy was linked with cognitive, structural and metabolic biomarker alterations we previously reported in this SCD cohort: Using correlation analyses we tested for associations between the volumes of the chBFN with the hippocampal gray matter volume, and posterior medial glucose consumption, and the trajectory of verbal memory performance. The SCD cases showed a significant total volume reduction of the chBFN, with largest effect sizes in the Ch1/2 and Ch4p subdivisions of the chBFN. The latter was associated with a reduced glucose metabolism in the precuneus for the SCD group only. These data show an early involvement of the cholinergic basal forebrain nuclei in SCD predominantly in Ch1/2 and Ch4p which supports the conceptual link between SCD and preclinical AD.

## Highlights

• Cholinergic forebrain shows atrophy in SCD.
• Ch12 and posterior part of Nucl. Basalis Meynert (Ch4p) show largest effect size.
• Atrophy of the Ch4p correlates with reduced glucose metabolism in precuneus.

## 1 Introduction

The research focus of early disease detection in Alzheimer's disease (AD) is moving to the preclinical stage, where cognitive performance levels are still in the normal range (Sperling et al., 2014; Sperling et al., 2011). Frequently, identification of these cognitively normal at-risk individuals is based on biological markers, either genetic risk factors (e.g. familial predisposition, or APOE epsilon 4 carriers), or by direct measurement of amyloid depositions using cerebral spinal fluid (CSF) assays or positron emission tomography (PET). Another, clinically-based approach is the examination of individuals who experience subjective cognitive decline (SCD) while their objective cognitive performance level is still in the normal range, which may reflect incipient AD (Jessen et al., 2014). Indeed, accumulating evidence suggests that SCD individuals are at an increased risk of future dementia (Dufouil et al., 2005; Glodzik-Sobanska et al., 2007; Jessen et al., 2010; Reisberg et al., 2010; van Oijen et al., 2007), and frequently display incipient alterations of AD-related neuroimaging biomarkers (Amariglio et al., 2012; Erk et al., 2011; Perrotin et al., 2012; Visser et al., 2009; Wang et al., 2013). For example, structural MRI investigations with SCD subjects show reduced volumes in areas that are typically affected in clinical AD, i.e. hippocampus and entorhinal cortex (Jessen et al., 2006; Saykin et al., 2006; Scheef et al., 2012; van de Pol et al., 2009). A recent study (Peter et al., 2014) observed that SCD patients did not only show a higher similarity of gray matter atrophy patterns with clinical AD patients than controls, but that SCD patients with more AD-like atrophy patterns showed stronger decline in longitudinal episodic memory tests. The same SCD population provided initial FDG PET evidence for reduced brain metabolism in the right precuneus (Scheef et al., 2012) which overlapped with the more extensive patterns of temporoparietal hypometabolism frequently observed in prodromal and clinical AD (Chetelat et al., 2003; Drzezga et al., 2003; Minoshima et al., 1997).

It is well established that AD is associated with cell loss of the cholinergic basal forebrain nuclei leading to a reduced cholinergic innervation of the cortex (Davies and Maloney, 1976; Lehericy et al., 1993; McGeer et al., 1984; Perry, 1980; Ruberg et al., 1990; Whitehouse et al., 1981). The cholinergic denervation contributes to the development of clinical symptoms and is the basis for the efficacy of acetylcholine esterase inhibitors in the treatment of cognitive impairment in AD (Dumas and Newhouse, 2011; Ellis et al., 2006; Furey, 2011).

MRI-based volumetry has shown that gray matter volume of the chBFN declines from prodromal to clinical stages of AD (Grothe et al., 2012; Kilimann et al., 2014), which is in line with neuropathological findings (i.e.(Lehericy et al., 1993)). While some extent of structural basal forebrain atrophy can also be observed in healthy aging, chBFN atrophy is more pronounced in individuals with early AD, especially in those showing a clinical progression over time (Grothe et al., 2013). The earliest affected part of the cholinergic system is the Nucleus basalis Meynert (NBM), especially the posterior part (Ch4p), showing volume reductions already in patients with mild cognitive impairment (MCI) as a supposed prodromal disease stage of dementia due to AD (Grothe et al., 2010; Kilimann et al., 2014). Furthermore, basal forebrain atrophy in MCI patients shows cross-sectional correlations with atrophy of other cortical brain regions implicated in AD, such as hippocampus and precuneus, as well as with alterations in connecting fiber tracts (Grothe et al., 2010; Teipel et al., 2011), which may reflect an early involvement of basal forebrain degeneration is AD pathogenesis. Actually, longitudinal data from the ADNI cohort show that NBM atrophy in mildly amnestic individuals precedes entorhinal degeneration and can predict longitudinal memory impairment (Schmitz et al., 2016).

Critically, a correlation of basal forebrain volumes with amyloid deposition in cognitively normal and mildly impaired non-demented elderly suggests that chBFN alterations may already be present in preclinical at-risk individuals with confirmed AD pathology (Grothe et al., 2010). Moreover, an earlier study found reduced basal forebrain volume in cognitively unimpaired subjects who subsequently developed dementia due to AD (Hall et al., 2008). Together, these previous findings suggest that atrophy of the basal forebrain is an early event in AD pathogenesis that is already emerging in preclinical AD. Thus, if SCD frequently reflects preclinical AD, we should expect similar incipient chBFN atrophy patterns.

In this study, we measured chBFN volumes in a previously reported sample of SCD subjects who predominantly report subjective memory impairment (Peter et al., 2014; Scheef et al., 2012). Based on high-resolution T1-weighted MRI data, we compared the chBFN volumes in SCD subjects with a matched group of elderly non-SCD individuals. We hypothesized that the SCD cohort will show reduced chBFN volumes, most pronounced in the posterior part Ch4p. To examine their relationship with other putative biomarkers of AD progression, we assessed the association of basal forebrain volumes with (a) cross-sectional and longitudinal memory performance over the course of up to 4 years (Peter et al., 2014), and with (b) right hippocampal volume reduction, and right precuneal hypometabolism (Scheef et al., 2012): In our previous report, these regions did not only show the most robust group differences, but also presented correlations with subsequent memory decline, making them the most plausible candidates for AD-related neurodegeneration in this sample.

We expected that this atrophy would be associated with metabolic and structural markers of AD-typical neurodegeneration, as well as with declining memory performance over time.

## 2 Materials and methods

### 2.1 Participants

Individuals with SCD (*n* = 24) were recruited by the memory clinic of the Clinical Treatment and Research Center for Neurodegenerative Disorders (KBFZ), Departments of Psychiatry and Neurology, University Hospital Bonn. The definition of SCD was based on the fact that subjects were referred to the memory clinic for work-up of memory complaints and on a standard question: ‘Do you feel like your memory is becoming worse?’ Possible answers to this question were: ‘no’, ‘yes, but this does not worry me’ and ‘yes, this worries me’. To be classified as SCD in this study, the subjects had to answer with ‘yes, this worries me’. We only included subjects whose memory worsening was confirmed by others (Vellas et al., 2008). In all cases, the informant was the spouse or a close relative (usually those who accompanied the patient with SCD during the visit of the memory clinic). In addition, only patients with an onset of experienced memory impairment within the last 10 years were included, in order to exclude life-long ‘memory complainers’.

The control cohort (*n* = 49) was recruited from the general population. Participants were included as controls if they denied any memory worsening or if a memory decline was only reported on active inquiry and was considered of no concern.

For all subjects, normal cognitive function was defined by performance on the Consortium to Establish a Registry for Alzheimer's Disease (CERAD) neuropsychological battery (Morris et al., 1989). For both groups, exclusion criteria were: (1) past or present neurological disorders; (2) significant medical diseases; (3) medication that may interfere with cognition, including any psychotropic medication; (4) any other detectable causes of memory impairment. Current and life-time psychiatric disorders were assessed with the German version of the Structured Clinical Interview for DSM-IV, SCID (Wittchen et al., 1997). Subjects with any psychiatric disorder at present or in the past were excluded, with the exception of a single depressive episode >10 years ago, which was reported by two patients with SCD and four subjects in the control group. In addition, the Beck Depression Inventory (BDI) was completed by all subjects (Beck et al., 1961) (Table 1).

**Table 1:** Baseline characterization.

	Controls	SCD	Difference
Sex, F/M	17/32	6/18	Χ^2^ = 0.70; *p* = 0.40; ns
Age, Years, mean ± SD	66 ± 7.2	67 ± 6.1	*t* = −0.72; *p* = 0.47; ns
Years of education, mean ± SD	15.0 ± 2.8	15.0 ± 3.6	*t* = 0.09; *p* = 0.93; ns
Beck Depression Inventory, mean ± SD	2.7 ± 2.8	6.3 ± 4.6	*t* = −3.59; *p* = 0.001
APOE4 allele carriers, n	11(22%)	7 (29%)	Χ^2^ = 0.33; *p* = 0.56; ns
MMSE, mean ± SD	29.3 ± 1.0	28.6 ± 1.1	*t* = 2.62; *p* = 0.012
CERAD Word List learning (max. 30), mean ± SD	22.7 ± 3.3	21.8 ± 3.3	*t* = 1.13; *p* =0.26; ns
CERAD Word List recall (max. 10), mean ± SD	7.9 ± 1.5	7.6 ± 1.7	t = 0,81; *p* = 0.42; ns
CERAD global score, mean ± SD^a^	98.2 ± 5.9	97.0 ± 6.4	*t* = 0.80; *p* = 0.43; ns
Follow up interval [month], mean ± SD	34.6 ± 11.2	35.5 ± 14.4	*t* = 0.29; *p* = 0.78; ns
No. follow-up visits, mean ± SD	3.2 ± 0.7	3.3 ± 0.9	*t* = 0.43; *p* = 0.67; ns

no Bonferroni correction applied.

Abbreviations: CERAD = Consortium to Establish a Registry for Alzheimer's Disease, MMSE = Mini-Mental State Examination, SCD = Subjective Cognitive Decline, SD = Standard Deviation, APOE = apolipoprotein E.

a The CERAD global score is obtained by summing the individual CERAD items with verbal fluency truncated at 24 and by adding a global correction factor for age, gender, and education. A score of 85 points is considered the cutoff between MCI and normal performance (Chandler et al.; Neurology, 2005).

### 2.2 Standard protocol approval and patient consent

The study protocol was approved by the ethics committee of the medical faculty of the University of Bonn. It was performed in accordance with the Declaration of Helsinki. All participants provided written informed consent.

### 2.3 Experimental neuropsychological battery

The neuropsychological test battery was designed to test verbal and visual memory, processing speed and executive functions, and was administered at baseline and at follow-up visits.

Verbal memory was assessed with the Verbal Learning and Memory Test (VLMT) (Helmstaedter et al., 2001), the German version of the Rey Auditory Verbal Learning Test (Rey, 1964). Visual memory was tested with the Rey Complex Figure test (Osterrieth, 1944). Processing speed and executive functions were tested with the Trail Making Test (TMT) A and B (Reitan, 1958) and with a semantic lexical 2-min verbal fluency task (Aschenbrenner et al., 2000). Because episodic memory is the most severely and earliest affected cognitive domain in MCI and AD, we focus in this paper on VLMT only (Rabin et al., 2009).

Both groups returned to the memory clinic for three follow-up visits including neuropsychological testing (see below). On average, the SCD group returned 14 ± 3.0 months, 32.5 ± 6.1 months, and 45.1 ± 5.6 months after baseline, while the control group returned after 15.5 ± 2.9 months, 33.3 ± 6.1 months, and 41.7 months. Twenty-two SCD subjects had at least one follow-up, 16 had two follow-up visits and 11 had three follow-up visits. Forty-five participants of the control group returned for at least one follow-up visit, 36 participants had two follow-up visits, and 15 had three follow-up visits.

### 2.4 MRI

Structural data were obtained from 24 SCD subjects and 49 controls. All scans were performed on a 3 Tesla MR system (Philips Achieva, Philips, Best, Netherlands), equipped with an 8-channel SENSE head coil. For each participant, four high-resolution T1-weighted data sets were acquired consecutively, using a 3D turbo field echo (TFE) sequence (SENSE reduction factors 2.5AP, 1.5RL, TE/TR/flip angle = 3.6 ms/7.6 ms/8°, field of view 256 × 256 mm^2^, matrix size 320 × 320, number of slices = 170, spatial resolution 0.8 × 0.8 × 0.8mm^3^).

#### 2.4.1 Preprocessing of MRI data

Preprocessing was performed using the VBM8 toolbox (C. Gaser, www.dbm.neuro.uni-jena.de/vbm8/). Preprocessing included realignment and averaging of each participant's four structural data sets, tissue class segmentation, and spatial normalization to MNI standard space using the high-dimensional registration algorithm DARTEL (Ashburner, 2007). The total amounts of GM, white matter (WM), and cerebrospinal fluid (CSF) were kept constant during the normalization by multiplying each voxel value of the tissue-class-specific probability map with the determinant of the Jacobian matrix of the local deformation matrix (Good et al., 2001). The individual probability maps for GM, WM and CSF derived from the normalization procedure were summed up to estimate the total intra-cranial volume (ICV).

Based on the finding from Scheef et al., 2012, we chose the brain region that showed the nominally largest effect size in local gray matter volume group difference and was associated with memory decline. The rationale for this approach was on one hand to increase the likelihood to reveal any existing association between those imaging markers and the cholinergic forebrain volumes, and on the other hand to be as close as possible to the clinical aspect of the SCD group, i.e. memory decline. The region that meets both conditions was the right hippocampus.

Region of interest analysis (ROI) of gray matter volume for the right hippocampus was performed following a protocol described previously (Scheef et al., 2012) calculating the volume using the normalized unsmoothed GM partition. The ROI templates were taken from the WFU Pick Atlas (Maldjian et al., 2003). The individual ROI volumes were calculated by adding together all voxel intensity values of the GM partition within a ROI. The ROI volumes were normalized by dividing the individual ROI volumes with the individual ICV and multiplying the result with the mean ICV.

#### 2.4.2 Volumetry of the cholinergic basal forebrain nuclei

**Fig. 1:**
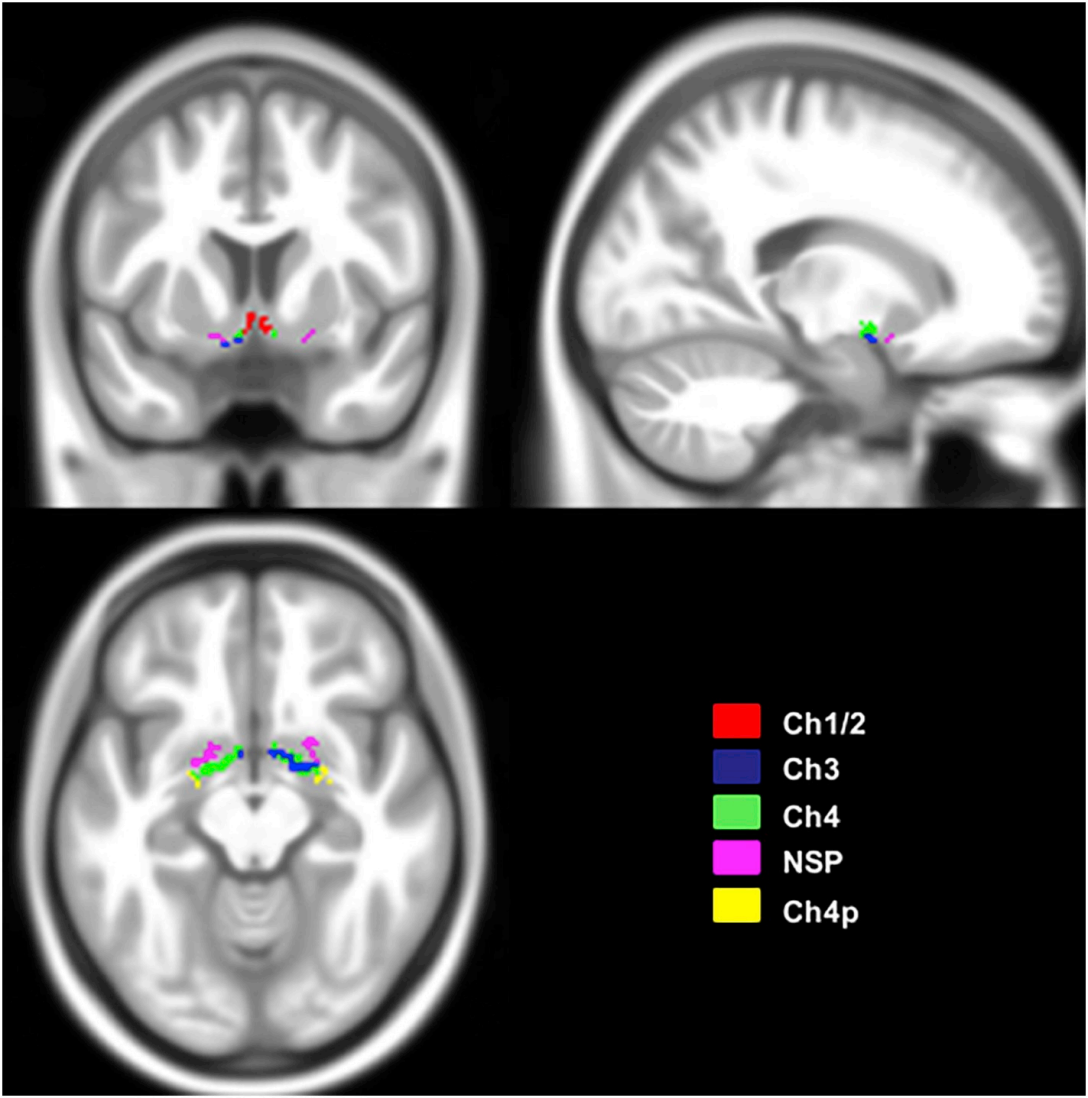
Outline of the anatomical position and extent of the cholinergic forebrain. Overlay of the cholinergic basal forebrain (chBFN) ROI's onto the T1-weighted MNI-template as described in detail by Kilimann et al., 2014. The different colors refer to the different nuclei. (For interpretation of the references to color in this figure legend, the reader is referred to the web version of this article.)

The cholinergic basal forebrain is composed of four different cell groups, which form partially overlapping ‘subunits’ (Ch1–Ch4), and project to different anatomical brain structures (Mesulam, 2004b; Mesulam and Geula, 1988; Mesulam et al., 1983a; Mesulam et al., 1983b). Ch1 reflects the medial septal nucleus, Ch2 the vertical nucleus of the diagonal band of Broca, Ch3 the horizontal limb of the diagonal band nucleus, and Ch4 refers to the cholinergic component of the nucleus basalis Meynert (NBM) (Mesulam, 2004b). The latter can be further subdivided into anterior lateral (Ch4al) and medial (Ch4am), intermediate (Ch4i), and posterior regions (Ch4p). A lateral extension of the Ch4al division has been termed Nucleus subputaminalis (NSP), or Ayala's nucleus, and has only been described in humans and anthropoid monkeys so far (Boban et al., 2006; Simic et al., 1999).The main cholinergic innervation of the hippocampus is provided by Ch1 and Ch2. Ch3 projects into the olfactory bulb, and Ch4 provides the cholinergic innervation of the cortex (Mesulam, 2004b; Mesulam et al., 1983a). Basal forebrain volumes were extracted using a cytoarchitectonic map of chBFN in MNI standard space (Kilimann et al., 2014). In short, the chBFN masks were derived from a post-mortem brain. This brain was scanned ‘in cranio’ as well as dehydrated before histologically processed. The histologically delineated chBFN subnuclei ROIs were then transferred onto the MRI of the dehydrated brain. The latter was transformed into the space of the in cranio MRI by applying a 12-parameter affine transformation and high-definition normalization and warped into MNI-space using DARTEL (Ashburner, 2007). The ROIs were transformed into the MNI-space by applying the derived transformation information on the chBFN ROIs in ‘dehydrated brain space’. An overview on the spatial location, and the extent of the chBFN in MNI-space is given in Fig. 1.

Due to the small size, Ch1 and Ch2 were not considered separately and will be referred to as ‘Ch1/2’ in the following. We also combined Ch4am, Ch4al without NSP, and Ch4i into one volume (Ch4). The posterior region (Ch4p) and NSP were considered separately, and the ROIs were combined across both hemispheres for all of the nuclei. As described for the hippocampal volume, the individual chBFN volumes were calculated by summing the voxel intensity values of the GM partition across the chBFN ROIs. The total volume of the basal forebrain nuclei (chBFN_tot_) was calculated by adding up the volumes of all subdivisions. All volumes were normalized by the ICV as described above.

### 2.5 FDG-PET

All [^18^F] fluoro-2-deoxyglucose PET (FDG-PET) scans were performed on a PET/CT scanner (Biograph, Siemens Medical Solutions, Inc.). Imaging started 30 min after injection of 150 MBq FDG. The acquisition started with low dose CT-scan for attenuation correction (19 mAs, 110 keV, 1 cm slice thickness). PET emission data acquisition was performed over 20 min. The subsequent attenuation correction for PET was based on a rescaling of the CT image and scatter correction using a single scatter model. Emission data were reconstructed with an in-plane spatial resolution of 1.3 × 1.3 mm^2^ and a slice thickness of 2.4 mm. The intrinsic resolution of the Biograph system is 9.3 mm (Prieto et al., 2010).

#### 2.5.1 Preprocessing of FDG-PET data

The preprocessing of the FDG-PET data was performed with SPM8; Wellcome Department of Cognitive Neurology, Institute of Neurology, London, UK. Data were transformed, normalized into a common stereotactic space, resliced with a voxel size of 2 × 2 × 2 mm^3^, and smoothed with a 12-mm kernel. Global counts were normalized by proportional scaling to a mean value of 50 mg/100 ml/min and by applying a threshold masking of 80% of maximum intensity. Following the approach described for the MRI-ROI selection, we chose the right precuneus as ROI for the PET analysis, because this region showed the highest effects strength in the direct group comparison and was associated with the memory decline over time (Scheef et al., 2012). The mean regional glucose metabolism for the right precuneus ROI was extracted, averaged, and additionally weighted by the mean ROI-GM volume for correlation analyses as described previously (Scheef et al., 2012). As for the MRI-analysis, the ROI templates were taken from the WFU Pick Atlas (Maldjian et al., 2003).

### 2.6 Statistical analysis

#### 2.6.1 Participants' characteristics and neuropsychological data at baseline

χ2 or *t*-test statistics were used to test for group differences in demographic data, CERAD data and in the neuropsychological battery. The main outcomes are displayed in the Table 1, Table 2.

**Table 2:** Neuropsychological test battery results at baseline.

	Controls		SCD		t-Statistic	
Test scores	Mean	SD	Mean	SD	t-Value	*p*-Value⁎
VLMT: immediate recall	53.2	7.7	48.3	11.5	1.88	0.068
VLMT: immediate recall after interference	10.9	2.6	9.4	3.9	1.74	0.091
VLMT: free delayed recall	10.5	3.2	9.4	4.0	1.41	0.163
VLMT: recognition	12.1	2.9	11.6	3.9	0.68	0.502
VLMT: memory total score	86.8	14.7	80.0	21.1	1.40	0.172
Rey-Complex Figures: recall	20.2	6.2	16.9	8.3	1.89	0.062
2 min letter verbal fluency	25.5	6.0	23.5	5.4	1.38	0.171
TMT A (letter s) [sec]	42,1	11.8	42.8	13.4	−0.21	0.834
TMT B (letter s) [sec]	95.4	35.5	103.8	58.1	−0.76	0.449

Abbreviations: SCD = Subjective Cognitive Decline, SD = Standard Deviation, VLMT = Verbal Learning Memory Test, TMT = Trail Making Test.

⁎ no Bonferroni correction applied.

#### 2.6.2 Modeling of the trajectories of memory performance

All subjects -except one SCD subject- participated in longitudinal neuropsychological follow-up. In this study, we focused on the VLMT performance only, which showed a significantly different trajectory for the groups, as reported previously (Peter et al., 2014), and whose decline is an established clinical predictor of AD (Bastin and Salmon, 2014). In order to characterize VLMT performance at T0 and follow-ups, the different VLMT sub-scores were reduced to one single factor (‘memory total score’) by an exploratory and confirmatory factor analysis of the four subscores (immediate recall, immediate recall after interference, delayed recall after 30 min, and recognition). To accommodate varying numbers of follow-up visits per subject and irregular interval length, growth curve modeling with linear growth factors was used to characterize the individual trajectory of the memory total score. The slope extracted by the growth curve model describes the individual course of memory performance over time. Both approaches, the factor analytical approach of the VLMT and the linear growth curve model, are described in detail elsewhere (Peter et al., 2014).

#### 2.6.3 Group comparison of the cholinergic forebrain nuclei volumes

To test whether the SCD group showed a decreased total volume of the basal forebrain nuclei (chBFN_tot_) we used a one-sided independent two sample t-test with the a priori hypothesis of smaller volumes in the SCD group. In a second step, we tested which subnucleus was affected and determined the respective effect sizes.

The Shapiro-Wilk test was used to test for normal distribution, and the Levene test was used to test for equality of variance (Levene, 1960; Shapiro and Wilk, 1965).

All further analyses were restricted to chBFN_tot_ and those nuclei showing a significant group effect.

#### 2.6.4 Exploratory correlation analysis between chBFN volumes and BDI-scores

As discussed below, groups differed significantly regarding BDI scores (see also: (Scheef et al., 2012)). To explore potential linear relationships of this confounded covariate with our variables of interest, we additionally investigated the correlations of the BDI with the cholinergic forebrain volumes for both groups separately.

#### 2.6.5 Correlation analysis between chBFN volumes and local GM volume

In order to investigate the association between chBFN volume reduction and our second putative marker of AD-related degeneration, we performed a correlation analysis in the SCD group between the significantly atrophied basal forebrain nuclei and the right hippocampus.

For any correlation yielding a significant effect, we conducted complementary analyses for the control group in order to test the specificity of the finding. Group differences were tested using linear regression analysis.

#### 2.6.6 Correlation analysis between chBFN volumes and FDG PET

To examine potential associations between chBFN volumes and regional glucose metabolism, similar analytic procedures as for the structural MRI data were applied. The right precuneus served as target region.

## 3 Results

### 3.1 Participant characteristics

All participants scored within 1.5 standard deviations (SD) of the mean according to age-, gender-, and education adjusted norms^2^ on all subtests of the CERAD test battery. Both groups did not differ in terms of gender, age, education, APOE 4 distribution (Hixson and Vernier, 1990, Table 1). Follow-up rates did not differ between the SCD and control group (χ^2^(3) = 2.67, not significant). No significant difference with regard to age, gender, and years of education existed between those participants still remaining in the study and those dropping out.

The SCD group scored slightly lower in the MMSE but was still well in the range of normal cognition (≥24 = no cognitive impairment; Tombaugh and McIntyre, 1992). The difference was not statistically significant after correcting for multiple comparisons. While a significant group difference was found in the BDI, both groups scored within the normal range, with no participant reaching clinical levels. As already reported in Scheef et al. (2012), we did not find any association between BDI scores and any cognitive measure or imaging marker. Especially for the cholinergic forebrain, correlations between BDI scores and chBFN_tot_ or its subnuclei were not statistically significant.

### 3.2 ChBFN-volume group differences

All chBFN volumes were normally distributed for both groups and homogeneity of variances was met according to Shapiro-Wilk and Levene's Test, respectively. The (normalized) chBFN_tot_ volume was smaller in the SCD group (mean ± SD = 600 ± 76 mm^3^) compared with the (normalized) volumes of the control group (mean ± SD = 642 ± 102 mm^3^). This difference of 42 mm^3^ was significant (t_71_ = 1.80, *p* = 0.038), with a small to moderate effect size (Cohen's d = 0.45).

Following up the significant overall effect, the Ch1/2 and Ch4p nuclei showed significantly smaller volumes in the SCD group as compared with the control group, with moderate effect sizes (81 ± 14 mm^3^ vs. 91 ± 19 mm^3^; t_71_ = 2.29, *p* = 0.013, d = 0.57 for Ch1/2, and 99 ± 15 mm^3^ vs. 106 ± 15 mm^3^, t_71_ = 2.01, *p* = 0.024, d = 0.50, for Ch4p, respectively).

The analyses for the other nuclei yielded no significant effect, although a smaller group difference in Ch3 volume approached significance (t_71_ = 1.61, *p* = 0.056, d = 0.38). The main results are summarized in Table 3.

**Table 3:** Normalized mean volumes of the cholinergic forebrain nuclei.

	Controls (n = 49)		SCD (n = 24)		Difference	Statistics		
(Sub)nucleus	mean [mm^3^]	SD [mm^3^]	mean [mm^3^]	SD [mm^3^]	[%]	t	p _one-sided_	d
Ch12	91.24	18.74	81.36	13.76	10.8	2.29	0.012⁎	0.572
Ch3	168.29	27.13	157.75	24.27	6.3	1.61	0.056	(0.384)
Ch4	141.99	26.15	134.08	20.15	5.6	1.30	0.098	–
Ch4p	106.36	15.94	98.56	14.85	7.3	2.01	0.024⁎	0.500
NSP	127.58	24.09	122.27	19.32	4.2	0.94	0.175	–
chBFN_tot_	642.42	101.86	600.17	75.59	6.6	1.80	0.038⁎	0.445

Abbreviations: SCD = Subjective Cognitive Decline, NSP = Nucleous subputaminalis = Ayala's nucleus, t = t-value, p = significance level, d = Cohen's d.

⁎ Statistical significant.

### 3.3 Memory performance and correlation with chBFN-volumes

Modeling memory performance over time using a growth curve model revealed a significant memory decline over time for the SCD group (slope_SCD_ = −0.033 ± 0.017), compared to a slight improvement for the control group (slope_Control_ = 0.150 ± 0.123). The group difference was significant (t_70_ = 10.2, *p* <0.000). Neither for the control group nor for the SCD cohort we found any significant correlations between chBFN volumes and the memory performance over time.

### 3.4 Correlation analysis between chBFN volumes and local GM volume

No significant correlation was found between Ch1/2 and right hippocampal volume (Table 4). For both groups, the Ch4p volume was highly correlated with the gray matter volume of the right hippocampus (Table 4). However, this correlation did not differ significantly between groups. In an explorative approach, we also correlated the individual Ch1/2 and Ch4p volumes with the individual total hippocampal volumes. This did not yield a different result. No significant correlation was found for Ch1/2 and hippocampal volumes but the correlation between the Ch4p the hippocampal volume was highly significant. As for the unilateral ROI, the correlation coefficients did not differ significantly (Control: r_Ch4p,hippo_ = 0.647, *p* <0.001; SCD: r_Ch4p, hippo_ = 0.656, *p* = 0.001; z = 0.06, *p* =0.9; all tests were performed two sided).

**Table 4:** Correlations between cholinergic forebrain nucleus volumes and regional gray matter volumes/regional glucose metabolism.

ROI		GM Volume		Glucose Metabolism	
		Right hippocampus		Right precuneus	
	Statistic	CTR	SCD	CTR	SCD
Ch1/2	**r**	0.108	0.005	−0.184	−0.103
	**p**	0.470	0.982	0.216	0.656
Ch4p	**r**	**0.501**	**0.526**	−0.190⁎	**0.635**⁎
	**p**	0.000	0.012	0.201	0.002

Abbreviations: CTR = Control; SCD = Subjective Cognitive Decline;

r = Pearson correlation coefficient; p = significance level (2-sided).

**BOLD**: significant correlations.

⁎ Significant group differences.

### 3.5 Correlation analysis between chBFN volumes and FDG PET

Ch1/2 did not show any significant correlations with metabolism in the respective ROI for any single group, or after pooling the data across both groups.

**Fig. 2:**
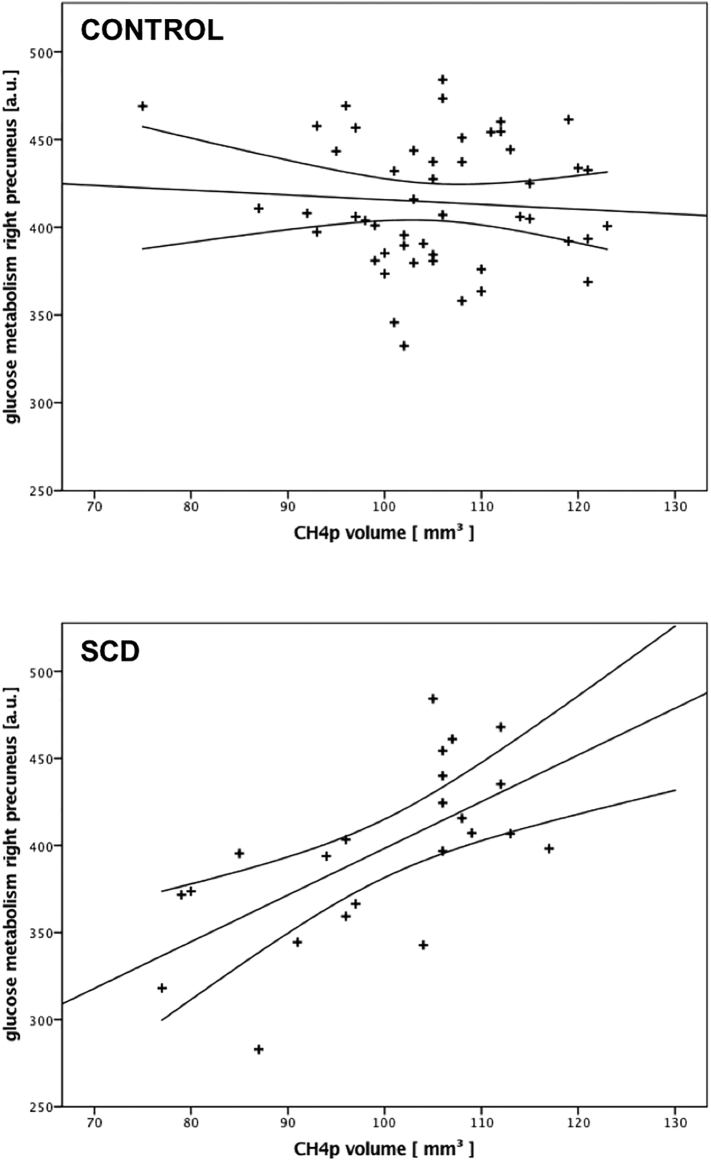
Correlation between the Ch4p volume and glucose metabolism. The glucose metabolism of the precuneus correlates significantly with the volume of the posterior part of the Nucleous basalis Meynert (Ch4p) only for the SCD group, whereas no (significant) correlation was found for the control group. This group difference was significant.

Meanwhile, Ch4p showed a significant association with right precuneus metabolism for the SCD group only (Table 4) and yielded a significant group effect (right precuneus: T_group_ = 3.739, *p* = 0.0004; see also Fig. 2 and Table 4). Following the same explorative approach described above for the hippocampus, the results were almost paralleled. Pooling the precuneus metabolism across both hemispheres did not reveal a significant correlation between Ch1/2 volume and metabolism for any of both groups. The same holds true for the correlation between Ch4p for the control group. The correlation between Ch4p volume and bilateral precuneus metabolism showed a strong trend towards significance (*p* =0.053). The difference of the correlation coefficient was significant (Control: r_Ch4p, precuneus_ = −0.153, *p* =0.3; SCD: r_Ch4p, precuneus_ = 0.418, p = 0.053; z = −2.14, *p* =0.03; all tests were performed two sided).

## 4 Discussion

In the present study, we used in vivo volume measurements of cholinergic forebrain nuclei in SCD and correlated the findings with memory trajectory over time, hippocampal volume and glucose metabolism of the precuneus. Consistent with the idea that SCD patients show a higher risk of preclinical, AD-related neuroimaging and cognitive alterations, we were able to show that the cholinergic basal forebrain volume was smaller in SCD compared to controls matched for age, gender and ApoE status, consistent with previous findings in MCI and AD dementia. Even though almost every nucleus showed atrophy, greater effect sizes were observed for Ch1/2 and Ch4p than for Ch3. While these atrophic changes were not predictive for the cognitive trajectory of the SCD patients, they were related to other AD-related neuroimaging markers that we previously found to be altered in this cohort (Scheef et al., 2012). We showed a significant association between the volume of the Ch4p subregion of the Nucleus basalis Meynert and right precuneal hypometabolism, a brain region that is known to show alterations already in early stages of AD development. Importantly, the latter association was only present in the SCD group.

The observed basal forebrain atrophy is in line with postmortem and in vivo studies in prodromal and clinical AD patients: Neuropathological data show that AD is associated with a loss of cholinergic neurons of the chBFN (Arendt et al., 1985; Vogels et al., 1990), and a consecutive loss of cortical cholinergic innervation (Geula and Mesulam, 1989). Neuronal loss seems to follow a specific pattern. It is most pronounced in the posterior parts of the NBM, especially Ch4p, whereas the rostral parts are less affected (Arendt et al., 1985; Doucette et al., 1986; Liu et al., 2015; Vogels et al., 1990; Wilcock et al., 1983). Using structural MRI it was not only possible to replicate the histopathological data in AD, showing an atrophy of the whole chBFN in AD dementia (Grothe et al., 2012; Kilimann et al., 2014), but to demonstrate that atrophic changes in the NBM (Schmitz et al., 2016) and most pronounced in its posterior subdivision (Grothe et al., 2013; Grothe et al., 2012; Grothe et al., 2014) are already detectable in MCI patients. Notably, recent data show that basal forebrain volume in cognitively normal individuals is negatively associated with brain amyloid load (Grothe et al., 2014), suggesting that incipient atrophy already develops in preclinical AD. Consistent with the idea that SCD frequently reflects preclinical AD, our findings may extend the latter observations towards SCD patients by also showing a significantly reduced Ch4p (and Ch1/2) volume in this cognitively normal sample.

A relationship between the Ch4p alterations in our SCD patients and AD-related neurodegenerative mechanisms is further supported by the observed correlation between Ch4p volume, and the glucose metabolism the right precuneus, which was only observed in the SCD group. The lower the volume of the Ch4p the lower the glucose consumption in the right precuneus. The exploratory data analysis using a bilateral precuneus ROI revealed a similar result. Even though the effect seems to be driven by the right precuneus a larger group size might be needed to further elucidate any laterality effect. However, the correlation between the Ch4p volume and the right precuneus metabolism is a remarkable finding, because lower precuneus glucose metabolism is known to be a highly sensitive and early marker for the development of AD (Minoshima et al., 1999; Minoshima et al., 1997). A detection of a decreased metabolism in the posterior cingulate (PC) and precuneus region was associated with a conversion rate of >90% within two years from MCI to AD (Chetelat et al., 2003; Drzezga et al., 2003; Minoshima et al., 1997). Even though it is tempting to assume that a reduced cholinergic innervation of the parietal lobe triggers the observed reduction of the glucose metabolism in the medial parietal cortex, it is not very likely that the reported relationship between metabolism and Ch4p deterioration is causal. The main cortical projection site of the Ch4p subdivision is assumed to lie in the superior temporal gyrus and temporal pole (Jones et al., 1976; Mesulam and Geula, 1988; Mesulam et al., 1983a), whereas the precuneus has shown to be mainly connected to anterior parts of the chBFN (Mesulam, 2004b; Parvizi et al., 2006).

The link may be indirect, i.e. depend on the mediation (or common causation) of unidentified additional brain mechanisms. Here, integration with connectivity-based methods like resting-state fMRI (e.g. Hafkemeijer et al., 2013; Verfaillie et al., 2018) may help to explore underlying system-level mechanisms.

While the observed associations of basal forebrain volumes with our second putative neuroimaging marker for AD neurodegeneration, right hippocampal GM volume, seem to provide further support for this claim at first sight, caution is warranted, since significant correlations were observed in both SCD and control participants: Accordingly, they may reflect a common non-AD process, e.g. aging.

Despite the agreement of the presented Ch4p/NBM atrophy in SCD with findings reported in MCI and AD, and the relation with local glucose metabolism, our data also show distinct differences regarding the role of Ch1/2 with respect to the literature. While reports on MCI and early-stage clinical AD published so far (Fujishiro et al., 2006; Grothe et al., 2012; Kilimann et al., 2014; Vogels et al., 1990) support the idea of a later spreading atrophy from the posterior to anterior parts of the cholinergic forebrain (Mesulam, 2004a), we did already find an early involvement of the Ch1/2 complex. Moreover, although the Ch1/2 volume was significantly smaller in the SCD group, it did not show an association with local glucose metabolism or local gray matter volume in AD-relevant brain regions. In summary, these Ch1/2 findings do not fit well with an AD-related neurodegenerative process, but may reflect non-AD pathologies, although CSF or PET biomarkers of amyloid (or other) neuropathology would be needed to further elucidate this issue (see: Limitations). On the other hand, Ch1/2 provides the main cholinergic innervation for the hippocampus which serves as a core structure for episodic memory, and Butler and colleagues have already shown that the volume of this region is predictive for memory performance in healthy adults (Butler et al., 2012). This is also confirmed by a recent study where Ch1/2 volume showed a much stronger association with memory performance than the other chBFN (Wolf et al., 2014). Therefore, findings of Ch1/2 atrophy in a population of SCD patients whose complaints were primarily characterized by symptoms of memory decline, show face validity. Yet, as we could not replicate the previously reported correlations between Ch1/2 atrophy and memory decline, functional interpretations have to be cautious.

### 4.1 Limitations

The reported data in this study are based on in vivo MRI measurements of structures that do not have a sufficient MR contrast to its neighborhood, and thus the derived chBFN volumes rely on a recently developed cytoarchitectonic atlas that was based on postmortem data of a single subject (Kilimann et al., 2014). Given that the ground truth is based on a single subject, the reliability of such an indirect measure can be called into question. Reliability is determined by two factors: 1. accurate definition of the ROIs, and (2) accuracy of the volumetric method, including GM segmentation and spatial normalization. Recent data show that the chBFN delineated based on the single subject template are in good agreement with chBFN delineated by a cytoarchitectonic probability map which was derived from 10 subjects (Grothe et al., 2010; Zaborszky et al., 2008). The center of gravity coordinates for the different chBFN of the Zaborszky probabilistic atlas project well onto the chBFN atlas published by Kilimann et al. (2014). Therefore, one can assume that the used atlas sufficiently represents the chBFN. As a consequence, the reliability is basically limited by the volumetric method itself. The consistent findings across imaging modalities, cohorts, in vivo and postmortem measurements in MCI and AD argue for the validity of the method. However, one should still keep in mind that this method provides an indirect volume measure only, which cannot distinguish between glial or neuronal loss, or between cell shrinkage or cell death. Considering the very small size of the chBFN, the method may also be more susceptible to unspecific normalization effects based on global group differences, but the employed high-dimensional normalization algorithm has been shown to reduce those effects to a minimum (Klein et al., 2009). The observed specificity of the atrophy pattern (showing the strongest differences for Ch1/2 and Ch4p), as well as the observed covariation with local GM volume in other brain regions further argues against unspecific normalization effects.

One might argue that groups differences in basal forebrain volumes were actually driven by confounding group differences in depression level, which represent an independent, non-neurodegenerative influence on SCD symptomatology. In general, all participants' BDI scores were within the normal range, and none of the participants reached clinical levels. Moreover, we did not find any significant correlation between the BDI scores and any cognitive measures or imaging marker (see: Scheef et al., 2012), including BF total and subregional volumes, making it is very unlikely that the group difference in BDI had any relevant influence on the study outcomes. In fact, there is a paucity of studies pointing towards basal forebrain volume reductions even in clinically depressed patients.

The sample size can be considered as an additional limitation. Even though the group sizes were sufficiently high to detect statistically significant differences in chBFN volumes, a larger group size might have allowed to elucidate further the role of the Ch1/2 atrophy within the SCD group. As an additional point one might argue that the tests used to compare the volume differences between both groups were one-sided and the reported data are not corrected for multiple comparisons. However, considering histology data and imaging data at preclinical stages, testing for atrophy only can be justified. In addition, the main findings of this paper, the atrophy of the cholinergic basal forebrain and significant correlation between Ch4p volume and precuneus hypometabolism in SCD, would still hold if one would only test for changes of the BF-total volume - ignoring the substructure of the cholinergic system- and perform the correlation analysis with those subregions showing the nominally largest volume reduction (i.e. Ch1/2–11%; Ch4p ~7%).

Also, the irregular numbers of follow-up visits and intervals in-between are not optimal. However, by using a growth curve model to determine the individual memory trajectory, we addressed this problem adequately. Unfortunately, the observation time was still too short to clearly associate the selected cognitive and neuroimaging parameters with individual conversion to dementia which would prove the underlying assumption that they represent actual biomarkers for preclinical AD progression. For this, the required observational time periods are estimated to be in the range of 10–15 years in samples that start at the SCD stage (Reisberg et al., 2008; Reisberg et al., 2010).

Of course, confirmation of preclinical AD in SCD patients does not require clinical long-term follow-up but could be achieved by in vivo measurements of AD pathology, i.e. CSF or PET examinations of amyloid burden. Unfortunately, this sort of information is not available for the present cohort. Yet, our initial findings may provide a starting point for further testing in larger-scale longitudinal study cohorts which also provide additional amyloid measures, e.g. the Harvard Aging Brain Study (Amariglio et al., 2015).

## 5 Conclusion

To the best of our knowledge, the presented data show for the first time that chBFN atrophy is already present in SCD which would support the idea of SCD as an at-risk population for preclinical AD. This notion is further supported by an association between Ch4p atrophy and decreased glucose metabolism in the right precuneus in the SCD group. These findings may provide a good starting point for larger-scale studies which also take into account direct measures of AD pathology, and longer follow-up durations to monitor the long-term clinical outcomes of this risk population.

## Footnotes

2 The German norms can be accessed at: www.memoryclinic.ch.
